# LONG-TERM BENEFITS OF REASONING TRAINING: A PREDICTED DIFFERENCE APPROACH

**DOI:** 10.1093/geroni/igz038.1618

**Published:** 2019-11-08

**Authors:** Michael Marsiske

**Affiliations:** University of Florida, Gainesville, Florida, United States

## Abstract

In 928 ACTIVE participants, we investigated predictors of exceptional reasoning performance ten years post-enrollment. Participants had been randomized into a training arm (memory, reasoning, or speed of processing) or a no-contact control group. Each participant received an age- and education adjusted expected normative trajectory on a reasoning composite score, derived from the untrained control group. They were then classified as within- (n=467, 50%), above- (n=285, 31%), or below-normative expectation (n=176, 19%) ten years post-training. At a p<.001 significance criterion, reasoning training (b=, 0.632, OR =1.88) and younger age (b=-0.048/year, OR = 1.05) were associated with 10-year above-normative expectations. No other baseline factors considered (other training arms, education, cardiovascular risk, life space, mobility, locus of control, morale, motivation) predicted ten year status, nor did they interact with training arm. Reasoning training appears to have produced long term alterations in reasoning trajectory for many participants.

